# Antioxidant, anticholinesterase and antibacterial activities of *Stachys guyoniana* and *Mentha aquatica*

**DOI:** 10.1080/13880209.2016.1238488

**Published:** 2016-12-07

**Authors:** Maria Ferhat, Ebru Erol, Khadidja Aya Beladjila, Yunus Çetintaş, Mehmet Emin Duru, Mehmet Öztürk, Ahmed Kabouche, Zahia Kabouche

**Affiliations:** a Département de chimie, Laboratoire d’Obtention de Substances Thérapeutiques (LOST), Université des frères Mentouri-Constantine, Constantine, Algeria;; b Faculty of Sciences, Department of Chemistry, Mugla Sıtkı Koçman University, Muğla, Turkey

**Keywords:** DPPH, ABTS+, β-carotene/linoleic acid, CUPRAC, metal chelating, cholinesterase inhibitory activity, agar dilution

## Abstract

**Context:***Stachys guyoniana* Noë ex. Batt. and *Mentha aquatica* L. are two Algerian Lamiaceae used in folk medicine.

**Objective:** To investigate their antioxidant, anticholinesterase and antibacterial activities.

**Material and methods:***n*-Butanol (BESG), ethyl acetate (EESG) and chloroform (CESG) extracts of *S. guyoniana* and methanol (MEMA) and chloroform (CEMA) aerial part extracts of *M. aquatica* and methanol (MERMA) and acetone (AERMA) roots extracts of *M. aquatica* were evaluated for their antioxidant activity by the β-carotene-linoleic acid, DPPH^•^ and ABTS^•^^+^ scavenging, CUPRAC and metal chelating assays. The anticholinesterase activity was tested against AChE and BChE. The antibacterial activity was assessed by MICs determination against *Escherichia coli, Staphylococcus aureus, Pseudomonas aeruginosa*, *Salmonella heidelberg*, *Klebsiella pneumoniae, Enterobacter aerogenes* and *Morganella morganii* strains.

**Results:** In the β-carotene test, the CESG (IC_50_: 2.3 ± 1.27 μg/mL) exhibited the highest activity. The BESG was the best scavenger of DPPH^•^ (IC_50_: 2.91 ± 0.14 μg/mL). In the ABTS test, AERMA was the most active (IC_50_: 4.21 ± 0.28 μg/mL). However, with the CUPRAC, the BESG exhibited the best activity (A_0.50_: 0.15 ± 0.05 μg/mL) and was active in metal chelating assay with 48% inhibition at 100 μg/mL. The BESG was the best AChE inhibitor (IC_50_: 5.78 ± 0.01 μg/mL) however, the AERMA showed the highest BChE inhibitory activity (IC_50_: 19.23 ± 1.42 μg/mL). The tested extracts exhibited a good antibacterial activity.

**Conclusion: **This study demonstrated good antioxidant, anticholinesterase and antibacterial potential of *S. guyoniana* and *M. aquatica,* which fits in well with their use in folk medicine.

## Introduction

*Stachys* (Lamiaceae) is a large genus that includes between 275 and 300 species (Bhattacharjee 1980; Mabberley 1997). This genus is mainly distributed in subtropical and tropical regions of both hemispheres (Piozzi et al. 2002). In Algeria, it is represented by 14 species including the endemic species *Stachys guyoniana* Noë ex. Batt. (Quezel & Santa 1963).

Some *Stachys* species are used in folk medicine to treat genital tumours, sclerosis of the spleen, inflammatory diseases, cough and ulcers (Zargari 1990), fevers, diarrhoea, sore mouth and throat, internal bleeding and weaknesses of the liver and heart (Conforti et al. 2009). In phytotherapy, tea made from the whole plant or leaves is used for its sedative, antispasmodic, diuretic and emmenagogue activity (Lewis & Elvin-Lewis 1977; Duke 1986). Extracts obtained from the aerial parts of *S. schtschegleevii* Sosn. ex Grossh have been used traditionally in North West of Iran for the treatment of infective, asthma, rheumatics and other inflammatory disorders (Mozaffarian 1982). *Stachys* species are reported to have many pharmacological activities including anti-inflammatory (Khanavi et al. 2005), anti-anxiety (Rabbani et al. 2003), antibacterial (Grujic-Jovanovic et al. 2004), anti-nephritic (Hayashi et al. 1994), anticancer (Amirghofran et al. 2006), anti-*Helicobacter* (Stamatis et al. 2003), antioxidant (Aydin et al. 2006) and cytotoxic effects (Haznagy-Radnai et al. 2008). In addition, many *Stachys* species are used in the preparation of food such as yoghurt or jelly to improve the taste and as flavours and seasoning (De Vincenci et al. 1997; Conforti et al. 2009). Decoctions or infusions of *Stachys* are applied as tonics to treat skin or taken internally for stomach disorders (Öztürk et al. 2009). The flavonoids are the most important secondary metabolites of reported *Stachys* (Karioti et al. 2011; Skaltsa et al. 2007). From reported works, plants of the genus *Stachys* may be considered as good sources of natural antioxidant for medicinal uses against aging and other diseases related to radical mechanisms (Tundis et al. 2014). Flavonoids, iridoids, fatty acids and phenolic acids have been identified as the main classes of secondary metabolites of species from this genus (Duru et al. 1999). The occurrence and the biological properties of diterpenoids from roots and aerial parts of the species of the genus *Stachys* was previously reviewed (Piozzi & Bruno 2011).

*Mentha* genus (Lamiaceae) includes approximately 30 species, plants of this genus are perennial and native to Europe, cultivated in the USA, Canada, Europe, Asia, Australia and South America (McKay & Blumberg 2006; Deschamps et al. 2008). Mint is valued for its multipurpose uses in the field of pharmaceuticals, cosmetics as well as for flavouring foods, beverages and tobacco (Coruh et al. 2007). The peppermint has many properties such as antimicrobial, antiviral, anti-inflammatory, mildly anaesthetic, antispasmodic, antiulcer, cytoprotective and hepatoprotective (Shah & Mello 2004). The dried leaves of *Mentha aquatica* together with those of *Tagetes minuta* L. (Asteraceae) are burned and the smoke inhaled by the Venda people of South Africa to treat mental illnesses (Arnold & Gulumian^1984^) and as a remedy against colds, respiratory problems and to protect against removal of “curses” and “evil spirits” (Pooley 2005).

Many scientists have focused on medicinal and edible plants to discover natural antioxidants to avoid toxic effects of synthetic antioxidants. The use of antioxidants may reduce the progression of Alzheimer’s disease (AD) and minimize neuronal degeneration (Atta-ur-Rahman & Choudhary 2001). Thus, the development and utilization of more effective antioxidants of natural origins as anticholinesterase compounds are desired (Gülsen et al. 2010). AD is frequently seen among elderly people all around the world. Some synthetic acetylcholinesterase inhibitors such as tacrine and donepezil have been used for the treatment of AD but they have several adverse effects (Mukherjee et al. 2007).

The present study deals with the antioxidant activity by using ABTS^^•^+ ^scavenging, DPPH^•^, CUPRAC assays, β-carotene, ferrous-ions chelating and anticholinesterase and antibacterial activities of the Algerian species *S. guyoniana* and *M. aquatica* which are investigated here, for the first time.

## Materials and methods

### Chemicals and instruments

A 96-well microplate reader, SpectraMax 340PC^384^, Molecular Devices (Sunnyvale, CA) was used for the antioxidant and anticholinesterase activities. Software PRO v5.2 (Sunnyvale, CA) was used to calculate the measurements. The absorbance was measured on a Thermo scientific 300-UV Spectrophotometer (Waltham, MA). The chemicals were obtained by Sigma-Aldrich GmbH, Sternheim, Germany.

### Plant material

The aerial parts of *S. guyoniana* and the whole parts of *M. aquatica* were collected in May 2012 at Constantine (North Eastern Algerian) and identified by Professor Gérard De Bélair, Faculty of Sciences University of Annaba, Algeria. Voucher specimen were deposited at the Herbarium of the Laboratory of Therapeutic Substances (LOST), University of Constantine (LOST Sg.05.12, LOST Ma.05.12, respectively).

### Preparation of crude extracts

Air-dried and powdered aerial parts of *S. guyoniana* (400 g) were macerated at room temperature in a methanol solution (70%). The extract was concentred under low pressure, diluted and filtered to remove chlorophyll, then successively extracted with petroleum ether, chloroform, ethyl acetate and *n*-BuOH. The evaporation of solvents in vacuum led to the respective dried extracts: PESG (0.3 g), CESG (1 g), EESG (2.8 g) and BESG (40 g).

Air-dried and powdered aerial parts of *M. aquatica* (200 g) were macerated separately with CHCl_3_, MeOH/CHCl_3_ (1:1) and MeOH during three days. Filtration and evaporation of solvents led to three respective dry extracts, CEMA (7 g), MCEMA (5.2 g) and MEMA (4.3 g).

The roots of *M. aquatica* (100 g) were extracted using a Soxhlet apparatus with acetone. The evaporation of the solvent under *vacuum* led to the AERMA extract (1.3 g). The plant residue was dried and further extracted with 70% MeOH at room temperature. The residue was filtrated and the methanol was evaporated *in vacuum* affording the MERMA extract (5 g).

### Antioxidant activity

#### DPPH free radical scavenging assay

The free radical-scavenging activity of the extracts was determined by the DPPH^•^ assay (Blois 1958). In its radical form, DPPH absorbs at 517 nm, but upon reduction by an antioxidant or a radical species its absorbance decreases. Briefly, a 0.1 mM solution of DPPH^•^ in methanol was prepared and 4 mL of this solution was added to 1 mL of sample solutions in methanol at different concentrations. Thirty minutes later, the absorbance was measured at 517 nm. Lower absorbance of the reaction mixture indicated higher free radical-scavenging activity. BHA and α-tocopherol were used as antioxidant standards to compare the activity. The results are given as 50% inhibition concentration in μg/mL (IC_50_).

$$\mathrm{DPPH}-\mathrm{scavenging}\;\mathrm{effect}\;\left(\%\right)=\frac{A_{\mathrm{Control}}-A_{\mathrm{Sample}}}{A_{\mathrm{Control}}}\times 100$$

where *A*_Control_ is the initial concentration of the DPPH^•^ and *A*_Sample_ is the absorbance of the remaining concentration of DPPH^•^ in the presence of the extract and positive control. BHT and α-tocopherol were used as antioxidant standards, for comparison of the activity.

#### β-Carotene/linoleic acid assay

The antioxidant activity of the samples was evaluated using the β-carotene-linoleic acid test system (Miller 1971). β-Carotene (0.5 mg) in 1 mL of chloroform was added to 25 μL of linoleic acid and 200 mg of Tween 40 emulsifier mixture. After evaporation of chloroform *in vacuum*, 100 mL of distilled water saturated with oxygen, were added by vigorous shaking. Aliquots of 4 mL of this mixture were transferred to different test tubes containing different concentrations of the sample. As soon as the emulsion was added to each tube, the zero time absorbance was measured at 470 nm using spectrophotometer. The emulsion system was incubated for 2 h at 50 °C. Ethanol was used as a control while BHA and α-tocopherol were used as antioxidant standards. The results were given as IC_50_ and were calculated from the graph of antioxidant activity percentage against sample concentration.

The bleaching rate (R) of β-carotene was calculated according to the following equation:

$$R\;=\;\frac{\ln a/b}{t}$$

where ln is natural log, *a* is absorbance at time zero, *b* is absorbance at time *t* (120 min). The antioxidant activity was calculated in terms of percent inhibition relative to the control, using the following equation:

$$\mathrm{Antioxidant}\;\mathrm{activity}\;\left(\%\right)=\frac{R_{\mathrm{Control}}-R_{\mathrm{Sample}}}{R_{\mathrm{Control}}}\times 100$$

#### ABTS cation radical decolorization assay

The ABTS^•+ ^scavenging activity was determined according to the method of Re et al. (1999). The ABTS^•+ ^was produced by the reaction between 7 mM ABTS in water and 2.45 mM potassium persulfate, stored in the dark at room temperature for 12 h. Before usage, the ABTS^•+^ solution was diluted to get an absorbance of 0.703 ± 0.025 at 734 nm with ethanol. Ethanol was used as a control, while BHA and α-tocopherol were used as antioxidant standards for comparison of the activity. The results were given as IC_50_. The sample concentration providing 50% ABTS^•+ ^scavenging effect (IC_50_) was calculated from the graph of ABTS^•+ ^scavenging effect percentage against sample concentration.

$${\mathrm{ABTS}}^{•+}\mathrm{scavenging}\;\mathrm{effect}\;\left(\%\right)=\frac{A_{\mathrm{Control}}-A_{\mathrm{Sample}}}{A_{\mathrm{Control}}}\times 100$$

where *A*_Control_ is the initial concentration of the ABTS^•+^ and *A*_Sample_ is the absorbance of the remaining concentration of DPPH^•^ in the presence of the extract and positive control. BHT and α-tocopherol were used as antioxidant standards for comparison of the activity.

#### Cupric reducing antioxidant capacity (CUPRAC)

The cupric reducing antioxidant capacity was determined according to the CUPRAC method (Apak et al. 2004). Results were recorded as absorbance compared with the absorbance of BHA and α-tocopherol, which were used as antioxidant standards.

### Ferrous ions chelating activity

The chelating activity of the extracts on Fe^2+^ was measured using Ferrin (Decker & Welch 1990) with slight modifications. The extracts solution (80 μL dissolved in ethanol in different concentrations) were added to 40 μL 0.2 mM FeCl_2_. The reaction was initiated by the addition of 80 μL 0.5 mM ferene. The mixture was shaken vigorously and left at room temperature for 10 min. After the mixture reached equilibrium, the absorbance was measured at 593 nm. The metal chelation activity was calculated using the following equation:

$$\mathrm{Metal}\;\mathrm{chelating}\;\mathrm{activity}\;\left(\%\right)=\frac{A_{\mathrm{Control}}-A_{\mathrm{Sample}}}{A_{\mathrm{Control}}}\times 100$$

where *A*_Control_ is the initial concentration devoid of sample and *A*_Sample_ is the absorbance of sample in the presence of the chelator. EDTA was used as antioxidant standard for comparison of the activity.

### Polyphenol content

The total polyphenolics was determined by the Folin–Ciocalteu method by the use of gallic acid as a standard (Singleton et al. 1999).

### Phytochemical screening

#### Shinoda's test for flavonoids

Three pieces of magnesium chips and few drops of concentrated HCl were mixed with 0.5 g of each investigated extract previously dissolved in ethanol. After few minutes, the appearance of pink, orange or red purple coloured scarlet indicated the presence of flavonoids (Trease & Evans 2002).

#### Ferric chloride test for flavonoids

Each extract (0.5 g) was boiled with distilled water, then, filtered. Few drops of 10% ferric chloride solution were added to 2 mL of the filtrate. A green–blue or violet colouration indicated the presence of flavonoids (Trease & Evans 2002).

#### Test for steroids and triterpenoids (Liebermann–Burchard test)

The extract (1 mg) which was dissolved in a few drops of chloroform was mixed to 3 mL of acetic anhydride and 3 mL of glacial acetic acid. The solution was then warmed, cooled under the tap water and then drops of concentrated sulfuric acid were added slowly. The formation of bluish–green colour indicated the presence of steroids and triterpenes (Sofowora 1993).

### Determination of anticholinesterase activity

Acetylcholinesterase and butyrylcholinesterase inhibitory activities of the extracts were measured, by slightly modifying the spectrophotometric method developed by Ellman et al. (1961). Electric eel AChE and horse serum BChE were used, acetylcholine iodide and butyrylthiocholine chloride were employed as substrates of the reaction. DTNB (5,5-dithio-bis(2-nitrobenzoic)acid) was used for the measurement of the anticholinesterase activity. Hundred and fifty microlitres of 100 mM sodium phosphate buffer (pH 8.0), 10 μL of sample solution, dissolved in ethanol at different concentration and 20 μL of AChE (5.32 × 10^−3^ U) or BChE (6.85 × 10^−3^ U) solution, were mixed and incubated for 15 min at 25 °C. Then, 10 μL of 0.5 mM DTNB were added. The reaction was then initiated by the addition of 10 μL of acetylcholine iodide (0.71 mM) or butyrylthiocholine chloride (0.2 mM). The hydrolyses of these substrates were monitored spectrophotometrically by the formation of the yellow 5-thio-2-nitrobenzoate anion, as the result of the reaction of DTNB with thiocholine, released by the enzymatic hydrolysis of acetylcholine iodide or butyrylthiocholine chloride, respectively, at a wavelength of 412 nm, utilizing a 96-wellmicroplate reader. The measurements and calculations were evaluated by using Soft-max PRO v5.2 software. Galantamine was used as a reference standard. The results were recorded as (IC_50_. The sample concentration, providing 50% enzyme inhibition (IC_50_), was calculated from the graph of enzyme inhibition percentage against sample concentration.

### Antibacterial activity

The extracts were individually tested against a range of bacteria, namely *Escherichia coli* ATCC 25922, *Staphylococcus aureus* ATCC 43300*, Pseudomonas aeruginosa* ATCC 27853, *Salmonella heidelberg* (HS), *Klebsiella pneumonia* (HS), *Enterobacter aerogenes* (HS) *and Morganella morganii* (HS). The reference strains were obtained from the Pasteur Institute (Algiers). The other strains (HS) were obtained from the laboratory of bacteriology, Benbadis Hospital, Constantine, using conventional methods. Susceptibility of the bacterial strains to *S. guyoniana* and *M. aquatica* extracts was investigated using minimum inhibitory concentration (MIC) method, according to the Clinical and Laboratory Standards Institute (NCCLS 1997). MICs were determined using agar dilution method at different concentrations of the tested extracts, included in Mueller–Hinton (MH) agar plates. The extracts were dissolved in ethanol to a final concentration of 512 μg/mL. This was serially diluted twofold with MH medium to obtain concentration of 0.5, 1, 2, 4, 8, 16, 32, 64 and 128 μg/mL. The essays were performed in triplicate and the results were expressed as their average. Ampicillin was used as a positive reference standard.

### Statistical analyses

All data on both antioxidant and anticholinesterase activity tests were the average of triplicate analyses. The data were recorded as mean ± standard error meaning (SEM.). Significant differences between means were determined by Student’s *t-*test, *p* values <0.05 were regarded as significant.

## Results and discussion

### Antioxidant properties

There are several methods for determining the antioxidant activity. In this study, antioxidant properties of *n*-butanol, ethyl acetate and chloroform extracts of *S. guyoniana* (BESG, EESG, CESG, respectively) and *M. aquatica* methanol and chloroform extracts from the aerial parts (MEMA, CEMA, respectively) and the methanol and acetone extracts from the roots of *M. aquatica* (MERMA, AERMA) were determined by five complementary tests, namely, the β-carotene-linoleic acid assay for lipid peroxidation activity, DPPH^•^, ABTS^•+ ^assays for radical-scavenging activity and CUPRAC and metal chelating methods. The results, except for CUPRAC assay, were recorded as IC_50_. As shown in Table 1, the antioxidant activity of tested extracts was compared with those of standards (α-tocopherol, BHA and EDTA). As known, the β-carotene bleaching method reveals the level of inhibition of lipid peroxidation. In this assay, *S. guyoniana* extracts (EESG and CESG**)** were more active than *M. aquatica* extracts (CEMA) but the most polar extracts of both plants (BESG, MEMA and MERMA) were not active, the highest activity was exhibited by the CESG (IC_50_: 2.3 ± 1.27 μg/mL) followed by the EESG (IC_50_: 3.15 ± 0.75 μg/mL). In DPPH^•^ assay, *M. aquatica* extracts were all inactive nevertheless, *S. gutoniana* exhibited an excellent activity where the BESG and EESG (IC_50_: 2.91 ± 0.14 and IC_50_: 5.53 ± 1.02 μg/mL, respectively) showed a higher activity than the standard α-tocopherol (IC_50_: 7.31 ± 0.17 μg/mL). The less active extract, the CESG (IC_50_: 35.76 ± 1.06 μg/mL), was better than the standard BHA (IC_50_: 45.37 ± 0.47 μg/mL). In the ABTS assay, *M. aquatica* was more active than *S. guyoniana* where AERMA was the most active (IC_50_: 4.21 ± 0.28 μg/mL) closely to the standard α-tocopherol (IC_50_: 4.31 ± 0.10 μg/mL), followed by the CEMA (IC_50_: 5.38 ± 0.14 μg/mL) whereas the best activity of *S. guyoniana* was exhibited by the CESG (IC_50_: 7.29 ± 0.23 μg/mL). However, in the CUPRAC assay, the BESG and the MERMA exhibited the best activity (A_0.50_: 0.15 ± 0.05 μg/mL and A_0.50_: 0.49 ± 0.04 μg/mL) which was higher than the standards, α-tocopherol (A_0.50_: 0.54 ± 0.01 μg/mL) and BHA (A_0.50_: 1.41 ± 0.7 μg/mL). Differently to the other assays, only the BESG was active in metal chelating assay with 48% inhibition at the concentration of 100 μg/mL, which is half lower than the inhibition exhibited by EDTA ferrous ions (96.5%), used as a standard. The EESG was not active despite its richness with polyphenols as well as the BESG (Table 2) which may suggest that the chelation depends on the nature of the flavonoids which are different in these extracts. *S. guyoniana* polar extracts BESG (354.91 ± 1.70 mg/g) and EESG (300.50 ± 0.90 mg/g) were found to be the highest polyphenol content which may be responsible for their highest DPPH scavenging effect contrarily to *M. aquatica* extracts (MEMA: 58.05 ± 2.20 mg/g and MERMA: 45.77 ± 0.80 mg/g), which were very less rich in polyphenols (Table 2). In addition to the total polyphenolic-content determined by Folin–Ciocalteu method, the phytochemical screening of all extracts, for their flavonoids and triterpenes content, were achieved according to the Shinoda and ferric chloride tests and Liebermann–Burchard test, respectively. The results showed that *M. aquatica* extracts are rich in steroids and triterpenoids which may explain their good activity in ABTS and CUPRAC assays (Table 3). From these phytochemical screening results, the richness of the CESG with steroids and triterpenoids may also be responsible for its highest ABTS scavenging effect than the BESG and the EESG.

**Table 1.** Antioxidant activity of *S. guyoniana* and *M. aquatica* polar extracts by the β-carotene–linoleic acid, DPPH^•^, ABTS^•+^, CUPRAC and metal chelating assays.^a^

Plant	Extract	β-carotene-linoleic acid assay	DPPH^•^ assay IC_50_ (μg/mL)	ABTS^•+^ assay	CUPRAC assay A_0.50_ (μg/mL)	Metal chelating assay % of inhibition at 100 μg/mL
*Stachys guyoniana* aerial parts	BESG	NA	2.91 ± 0.14	29.08 ± 1.29	0.15 ± 0.05	48.00 ± 1.71
	EESG	3.15 ± 0.75	5.53 ± 1.02	21.57 ± 1.43	2.28 ± 0.01	NA
	CESG	2.30 ± 1.27	35.76 ± 1.06	7.29 ± 0.23	3.85 ± 0.20	NA
*M. aquatica* aerial parts	MEMA	NA^b^	NA	68.99 ± 0.15		NA
	CEMA	8.69 ± 0.7	NA	5.38 ± 0.14	NA	NA
*M. aquatica* roots	MERMA	NA	NA	51.07 ± 0.85	0.49 ± 0.04	NA
	AERMA	9.28 ± 0.32	NA	4.21 ± 0.28		NA
Standards^c^	α-Tocopherol	2.10 ± 0.08	7.31 ± 0.17	4.31 ± 0.10	0.54 ± 0.01	
	BHA	1.34 ± 0.04	45.37 ± 0.47	4.10 ± 0.06	1.41 ± 0.7	
	EDTA					96.5 ± 1.4

a IC_50_ values represent the means ± SEM of three parallel measurements (*p* < 0.05).

b Not active.

c Reference compounds: BHA: butylated hydroxyl anisole; EDTA: ethylenediaminetetraacetic acid.

**Table 2.** Total polyphenolic content of *S. guyoniana* and *M. aquatica* polar extracts.

Plant species	Extract	Total phenolic content (mg/g)^a^
*S. guyoniana* aerial parts	BESG	354.91 ± 1.70
	EESG	300.50 ± 0.90
*M. aquatica* aerial parts	MEMA	58.05 ± 2.20
*M. aquatica* roots	MERMA	45.77 ± 0.80

a mg/g gallic acid equivalent.

**Table 3.** Phytochemical screening of *S. guyoniana* and *M. aquatica* extracts.

Extract	Flavonoids Shinoda and ferric chloride tests	Steroids and triterpenoids Liebermann–Burchard test
BESG	++	+
EESG	++	+
CESG		++
MEMA		++
CEMA		++
MERMA		++
AERMA		++

### Anticholinesterase activity

As shown in Table 4, only the BESG and the AERMA exhibited an AChE inhibitory activity. The BESG activity (IC_50_: 5.78 ± 0.01 μg/mL) was a little less active than galantamine (IC_50_: 5.01 ± 0.10 μg/mL), used as a standard drug against AD. However, the AERMA, the CEMA and the BESG inhibited the BChE (IC_50_: 19.23 ± 1.42 μg/mL, IC_50_: 20.7 ± 2.11 μg/mL, IC_50_: 39.1 ± 1.41 μg/mL, respectively), better than the standard (IC_50_: 53.90 ± 0.56 μg/mL). It’s noteworthy that *M. aquatica* extracts (AERMA and CEMA), which are rich in steroids and triterpenoids, were the highest BChE inhibitors while the BESG, which is rich with polyphenols, was the best AChE inhibitor which demonstrates that the inhibition mechanisms of the two enzymes are different.

**Table 4.** Acetylcholinesterase (AChE) and butyrylcholinesterase (BChE) inhibitory activiy of *S. guyoniana* and *M. aquatica* extract.^a^

Plant	Extract	AChE assay	BChE assay
IC_50_ (μg/mL)			
*S. guyoniana* aerial parts	BESG	5.78 ± 0.01	39.10 ± 1.41
	EESG	NA^b^	NA
	CESG	NA	NA
*M. aquatica* aerial parts	MEMA	NA	NA
	CEMA	NA	20.18 ± 0.55
*M. aquatica*roots	MERMA	NA	NA
	AERMA	20.7 ± 2.11	19.23 ± 1.42
Standard	Galantamin	5.01 ± 0.10	53.90 ± 0.56

a Values expressed are means ± SEM of three parallel measurements (*p* < 0.05).

b Not active.

Previously, Gholamhoseinian et al. (2009) reported that *S. inflate* Benth. and *S. lavandulifolia* Vahl. could be used in the treatment of AD. The hexane extract of *S. lavandulifolia* showed the highest AChE inhibitory activity with an IC_50_ value of 13.7 μg/mL while the dichloromethane extract was the most active against BChE (IC_50_ value of 143.9 μg/mL) (Tundis et al. 2015). Previous works reported that essential oils and extracts, rich in terpenes, exhibited a strong AChE and BChE inhibitory activity (Tundis et al. 2015).

### Antibacterial activity

Table 5 shows the MICs which were performed by the serial dilution method (Agar method). Ampicillin was used as a positive control in these tests. The highest antibacterial activity of the BESG was observed against *S. aureus* ATCC 43300 and *E. aerogenes* (HS) with 32 ± 0.90 μg/mL and 32 ± 0.70 μg/mL MICs, respectively, whereas *E. coli* ATCC 25922 was the most inhibited strain by the CESG with 64 ± 0.60 μg/mL MIC value. However, the MEMA exhibited the best antibacterial activity (64 ± 1.40 μg/mL) against *M. morganii* (HS) whereas *S. heidelberg* (64 ± 0.70 μg/mL) and *K. pneumoniae* (64 ± 1.00 μg/mL) were the most inhibited strains by the MERMA.

**Table 5.** Antibacterial activity (MIC’s) of *S. guyoniana* and *M. aquatica* extracts.

	MIC (μg/mL)^a^				
Microorganisms	BESG^b^	CESG^b^	MEMA^b^	MERMA^b^	Ampicillin^c^
*E. coli* ATCC 25922^d^	128 ± 1.20	64 ± 0.60	–	128 ± 0.40	8 ± 0.40
*S. aureus* ATCC 43300^d^	32 ± 0.90	128 ± 1.10	128 ± 1.30	128 ± 0.80	4 ± 0.10
*P. aeruginosa*ATCC 27853^d^	–	–	128 ± 0.50	128 ± 1.60	–
*S. heidelberg* (HS)^e^	128 ± 1.10	128 ± 0.80	128 ± 0.90	64 ± 0.70	–
*K. pneumoniae* (HS)^e^	128 ± 1.00	128 ± 0.60	128 ± 2.00	64 ± 1.00	32 ± 0.40
*E. aerogenes* (HS)^e^	32 ± 0.70	128 ± 1.00	128 ± 1.40	128 ± 0.80	32 ± 0.60
*M. morganii* (HS)^e^	–	–	64 ± 1.40	128 ± 0.90	–

a Values are mean ± SD (*n* = 3).

b 128 μg/mL.

c 30 μg/mL.

d Referenced strain (Pasteur Institute-Algiers).

e Hospital strain (clinically isolated, CHU-Constantine).

## Conclusion

The results presented in this study are the first information on the antioxidant, anticholinesterase and antibacterial activities of two species, *S. guyoniana* and *M. aquatica*, collected from Constantine-Algeria. The antioxidant potential of various extracts of these plants, by the use of five complementary methods namely, β-carotene-linoleic acid, DPPH^•^ and ABTS^•+ ^scavenging, CUPRAC and metal chelating assays have shown that the polar extracts of *S. guyoniana* (BESG and EESG) which are rich in polyphenols possess the most DPPH antiradical effect while the apolar extracts of *M. aquatica,* which are rich in steroids and triterpenes, exhibited the best activity with ABTS and CUPRAC assays. Only the BESG was active in metal chelating assay which suggests that the mechanism depends on the nature of the flavonoids content. The less polar extracts of *M. aquatica* were the highest BChE inhibitors while the BESG was the best AChE inhibitor suggesting different inhibition mechanisms of the two enzymes. The investigated extracts inhibited mildly the growth of tested bacterial strains with MICs ranged between 32 and 128 μg/mL.

From our results and reported works, *S. guoniana* and *M. aquatica* could be considered as sources of antioxidants and antibacterial and may offer a treatment for Alzheimer’s disease.
